# Electrophysiological Evidence of Auditory and Cognitive Processing Deficits in Parkinson Disease

**DOI:** 10.1155/2021/6610908

**Published:** 2021-06-18

**Authors:** Robert L. Folmer, Jay J. Vachhani, Amy Riggins

**Affiliations:** ^1^National Center for Rehabilitative Auditory Research, VA Portland Medical Center, Portland, Oregon, USA; ^2^Department of Otolaryngology, Oregon Health & Science University, Portland, Oregon, USA; ^3^Department of Communication Sciences & Disorders, University of Wisconsin, Madison, USA

## Abstract

**Background:**

Parkinson's disease (PD) patients are at increased risk for central auditory processing (CAP) deficits and cognitive dysfunction. However, behavioral assessments of CAP and cognitive processing used in a previous study by our research team found few significant differences in performance between early-stage PD patients and age-matched control subjects. The objective of this study is to use auditory event-related potentials (AERPs) to compare CAP and cognitive functions in a population of PD patients with a group of age-matched control subjects.

**Methods:**

AERPs in response to tonal and speech stimuli were recorded from 35 adults who had a medical diagnosis of PD (23 males and 12 females; mean age = 66.9 ± s.d.11.2 years), and 35 age-matched control subjects who did not have PD or any other neurological disorders (31 males and 4 females; mean age = 65.4 ± s.d.12.3 years). Auditory stimuli included pure tones (500 and 1000 Hz) to elicit the P300 response and a dichotic digits paradigm to elicit the N200 processing negativity.

**Results:**

Compared to control subjects, PD patients exhibited significantly longer latencies of P300 and N200 components and smaller amplitude N200 components. Latency and amplitude of the N200 component were significantly correlated with participants' age. N200 amplitude was correlated with results from the Rey Auditory Verbal Learning Test (RAVLT) of cognitive ability. Latency of the P300 and amplitude of the N200 components were significantly correlated with results from the Spatial Release From Masking (SRM) behavioral CAP assessment.

**Conclusions:**

AERP assessments used in this study appear to be sensitive indicators of CAP and cognitive deficits exhibited by early-stage PD patients. While few significant differences in performance on behavioral CAP and cognitive tests were previously observed between this population of PD patients and age-matched control subjects, N200 and P300 components recorded in the present study revealed impaired neural processing by the PD group.

## 1. Introduction

Aging and hearing loss both contribute to declines in one's ability to process auditory information. For example, older people and those with hearing loss often have difficulty understanding conversational speech when background noise is present [1–3]. The average patient age for Parkinson's disease (PD) onset is approximately 60 years, and the prevalence of PD increases with age [4]. Since the majority of people 60 years old and older have significant hearing loss (HL), and the prevalence of HL increases with age [5, 6], a majority of PD patients have significant HL that will worsen over time. Patients with untreated hearing loss dedicate more of their resources to auditory perceptual processing to the detriment of other cognitive processes. Therefore, hearing loss may contribute to dementia through exhaustion of cognitive reserves, social isolation, sensory deafferentation, or a combination of these mechanisms [7]. Since cognitive decline and dysfunction are common sequelae of PD [4], untreated hearing loss is likely to exacerbate cognitive deficits that are experienced by many PD patients.

Guehl et al. [8], Lewald et al. [9], and Vitale et al. [10] reported that PD patients exhibited poorer performance on behavioral assessments of auditory processing compared to groups of age-matched, healthy control subjects. Therefore, the neuropathological mechanisms of PD appear to contribute to central auditory processing (CAP) deficits in this population [11]. A previous study by our research group also reported abnormal performance by a group of PD patients on behavioral CAP tests [12]. However, because most of these PD patients were in the early and less severe stages of disease, their performance on many CAP assessments did not differ significantly from that exhibited by a group of age-matched healthy control subjects.

The goal of the present study was to use auditory event-related potentials (AERPs) to assess CAP and cognitive functions in a population of PD patients and compare the results with a group of age-matched control subjects without PD or other neurological disorders. We hypothesized that electrophysiological recordings are more sensitive detectors of auditory and cognitive processing deficits exhibited by PD patients than are the behavioral assessments used in our previous study [12].

## 2. Methods

All procedures for the conduct of the study adhered to the requirements of the Institutional Review Board at VA Portland Medical Center, where the study was conducted.

Participants included 35 adults who had a medical diagnosis of PD and 35 age-matched control subjects who did not have PD or any other neurological disorders. All of the participants in this study were the same as those in our 2017 publication [12]. Also, the electrophysiological data reported in this article were collected around the same time as behavioral data previously reported for this population [12].

After written informed consent was obtained, participants underwent the procedures and assessments described below over the course of three sessions.

### 2.1. Assessments of PD Severity

The Hoehn and Yahr [13] and Schwab and England [14] scales were used to assess the stage and severity of PD for individuals in the patient group.

Parkinson patients were also asked to rate their abilities “during the past week” for 12 activities such as swallowing, handwriting, dressing, hygiene, falling, salivating, turning in bed, walking, and cutting food (these questions were taken from Part II of the Unified Parkinson's Disease Rating Scale [15]).

### 2.2. Neuropsychological Evaluation

The Rey Auditory Verbal Learning Test (RAVLT) [16] was administered to all study participants. This test evaluates a variety of functions: short-term auditory-verbal memory, rate of learning, learning strategies, retroactive and proactive interference, presence of confabulation or confusion in memory processes, retention of information, and differences between learning and retrieval. Participants are given a list of 15 unrelated words repeated over five different trials and are asked to repeat them. Another list of 15 unrelated words is given, and the subject must then repeat the original list of 15 words; this process is repeated again 30 minutes later.

### 2.3. Comprehensive Audiometric Evaluation

Pure tone air and bone conduction thresholds were measured in each ear using procedures recommended by the American Speech-Language-Hearing Association [17].

### 2.4. Assessments of Central Auditory Processing (CAP)

Several different CAP evaluations were administered to study participants: Staggered-Spondaic-Word (SSW) test [18], Masking-Level Difference (MLD) Test [19, 20], Gap in Noise (GIN) Detection Test [21], Dichotic Digits Test [22], Spatial Release From Masking (SRM) tests [23], and Speech Intelligibility in Noise was assessed using the Words in Noise (WIN) test [24]. These tests are described in our previous publication involving this study population [12].

### 2.5. Electrophysiological Recordings of Auditory Event-Related Potentials (AERPs)

Long-latency AERPs were recorded from 15 Ag/AgCl scalp electrodes using a Quik Cap and a Neuroscan EEG/EP system (Compumedics, Charlotte, NC) according to parameters described in Papesh et al. [25]. Responses to 50 repetitions of each stimulus were averaged to create individual responses within each condition. Grand average responses were created for each condition and group.

Latency and amplitude values of N100, P200, N200, and P300 components were determined by the agreement of the three judges. Long-latency AERPs were recorded from each subject in response to the following stimuli which were presented via Etymotic ER-3 insert earphones (Etymotic Research, Inc.; Elk Grove Village, IL).

#### 2.5.1. “Oddball” Paradigm (Tones) to Elicit P300 Responses

Stimuli: 50 1000-Hz tones (100 msec duration) and 200 500-Hz tones (100 msec duration) were presented in a randomized sequence at 85 dB SPL to each ear monaurally; target = 1000 Hz tone. Task: subjects counted higher-pitched (1000 Hz) tones silently to themselves. This electrophysiological protocol provides an objective measure of central auditory processing and cognitive function.

#### 2.5.2. Dichotic Digits

Stimuli: an assortment of 300 spoken digits (50 each: 1, 2, 3, 4, 5, or 6) delivered dichotically and presented randomly. Task: subjects pushed a button with their right index finger when they heard the target “four” in either ear. Because a study by Lewald et al. [9] reported that PD patients exhibited deficits in behavioral dichotic listening tasks, this electrophysiological protocol was included to provide an objective measure of neural processing and cognitive function during such a task.

### 2.6. Data Analysis

Mean and standard deviation values were calculated for each assessment, AERP component, and study group. Between-group comparisons were conducted using 2-tailed *t*-tests and applying appropriate Bonferroni corrections as needed. Pearson correlation calculations were also made in certain instances as indicated in the Results section. Tests of normality and homogeneity of variances confirmed the statistical appropriateness of these analyses for the data collected.

## 3. Results

### 3.1. Participant Characteristics

The PD group consisted of 35 adults (23 males and 12 females; mean age = 66.9 ± s.d.11.2 years). The control group also consisted of 35 adults (31 males and 4 females; mean age = 65.4 ± s.d.12.3 years) who had no history of PD or other neurological disorders. The difference in the proportion of males vs. females in each of the study groups was statistically significant (*p* < 0.03). Levodopa use by PD patients: all PD patients except one used levodopa medication daily—he had not yet started using this medication. Of 105 total appointments, PD patients reported that they were “on” the effects of levodopa for 95 appointments, “off” for 7 appointments, and “in-between” for 3 appointments.

For additional details about participant characteristics, see our previous publication involving this study population [12].

#### 3.1.1. Ratings of Daily Activity Abilities

For PD patients in this study, the total score on these 12 questions ranged from 3 to 27 (mean = 12.1 ± s.d.5.0), with higher scores indicating greater difficulty on the collection of tasks. These data, combined with Hoehn and Yahr and Schwab and England results, suggest that the majority of PD patients in this study were in the early—or less severe—stages of the disease.

### 3.2. Neuropsychological Evaluation

Mean RAVLT scores (total of trials 1 through 5) were 42.9 ± 7.8 for PD patients and 46.0 ± 11.5 for control subjects, which indicates normal performance for the age and education level of study participants [26]. The difference in mean scores between study groups was not statistically significant. Also, there was no statistically significant difference in the number of intrusions or repetitions made by the two study groups on this test.

### 3.3. Pure Tone Audiometry

Pure-tone audiometry indicated that both the control and PD groups had sloping, high-frequency sensorineural hearing loss which is typical for their age range (see Figure 1 in Folmer et al. [12]). Compared to age-matched healthy control subjects, PD patients exhibited worse hearing sensitivity for 1500 and 2000 Hz test frequencies. Pure tone average (PTA) threshold was 28.9 ± 14.1 dB HL for the control group and 33.9 ± 13.0 dB HL for the PD group.

We subdivided study participants into younger (≤65 years) and older (>65 years) groups according to age criteria used by da Silva Lopes et al. [27]. This grouping resulted in 19 younger (mean = 60.4 years) and 16 older (mean = 71.4 years) control subjects; 17 younger (mean = 60.5 years) and 18 older (mean = 71.5 years) PD patients. In general, hearing sensitivity for older participants (older controls = OC; older Parkinson = OP) was worse compared to younger participants (younger controls = YC; younger Parkinson = YP), especially for higher frequencies. Pure tone hearing thresholds did not differ significantly between the OC and OP groups. However, the younger PD group exhibited worse hearing between 1500 and 2000 Hz compared to the younger control group. This difference was the major contributor to overall differences in hearing sensitivity between the entire PD and control groups.

### 3.4. Assessments of Central Auditory Processing

As reported in our previous publication involving this study population [12], both study groups exhibited deficits in many assessments of central auditory processing (CAP), with PD patients performing significantly worse than the control group on the Spatial Release from Masking (SRM) 45° test condition only. Compared to the control group performance on the SRM test, the PD group had greater difficulty understanding sentences in a background of competing speech, and they also showed less improvement on this task when the target and competing sentences were separated in space (the 45° condition).

### 3.5. Electrophysiological (AERP) Results

#### 3.5.1. “Oddball” Protocol to Elicit the P300 Response

**Figure 1:**
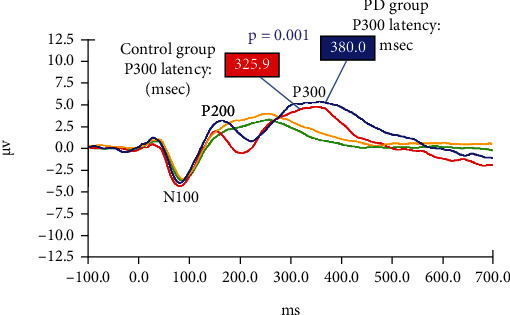
Grand averaged AERPs from both study groups in response to target (1000 Hz) and nontarget (500 Hz) tones during the “oddball” paradigm. Red: control group response to target tones; Green: control group response to nontarget tones; Blue: PD group response to target tones; Yellow: PD group response to nontarget tones.

As shown in Figure 1, AERP grand averaged responses to the “nontarget” 500 Hz tones (recorded at electrode Cz) were basically the same for PD patients and control subjects. The latency and amplitude of the N100 component are not significantly different between the study groups. However, the mean latency of the P300 component in response to the “target” 1000 Hz tones was significantly delayed (*p* = 0.001) for PD patients (380.0 ± 44.8 msec) compared to control subjects (325.9 ± 89.5 msec). There was no statistically significant difference in P300 amplitude between the PD and control groups.

**Figure 2:**
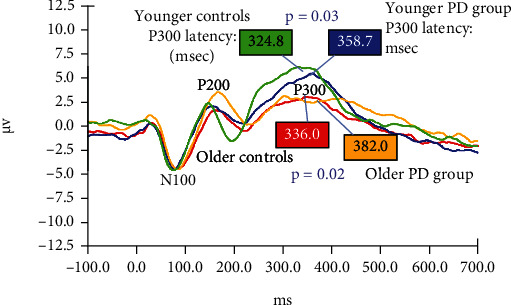
Grand averaged AERPs from study subgroups (sorted by age) in response to target (1000 Hz) tones during the “oddball” paradigm. Red: older control group; Green: younger control group; Blue: younger PD group; Yellow: older PD group response.

Figure 2 shows grand averaged responses to the 1000 Hz target tone for all four study subgroups by age (YC, YP, OC, and OP). The mean latency and amplitude of the N100 component are not significantly different among the subgroups. However, P300 amplitude is greater for younger participants (YC and YP) compared to the older subgroups (OC and OP). Also, the latency of the P300 is significantly greater for PD patients compared to control subjects in both the younger (mean = 324.8 vs.358.7 msec; *p* = 0.03) and older (mean = 336.0 vs.382.0 msec; *p* = 0.02) subgroups.

#### 3.5.2. Dichotic Digits Stimuli

**Figure 3:**
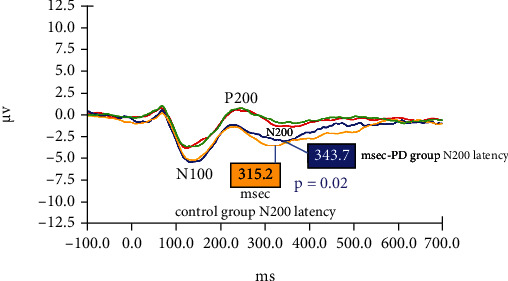
Grand averaged AERPs from both study groups in response to target (4) and nontarget (1,2,3,5,6) digits during the dichotic digits paradigm. In these trials, the target digit (4) was presented to the right ear only. Red: control group response to nontarget digits; Green: PD group response to nontarget digits; Blue: PD group response to target digits; Yellow: control group response to target digits.

**Figure 4:**
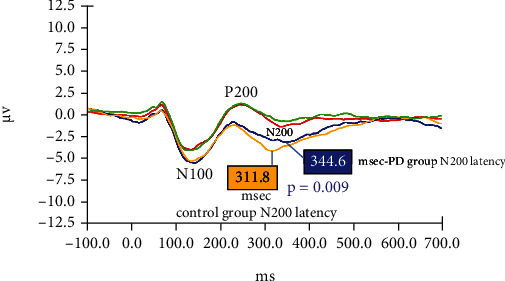
Grand averaged AERPs from both study groups in response to target (4) and nontarget (1,2,3,5,6) digits during the dichotic digits paradigm. In these trials, the target digit (4) was presented to the left ear only. Red: control group response to nontarget digits; Green: PD group response to nontarget digits; Blue: PD group response to target digits; Yellow: control group response to target digits.

Figure 3 shows grand averaged AERPs from both study groups in response to target stimuli (digit 4) in the right ear and nontarget stimuli (digits 1, 2, 3, 5, 6).  Figure 4 shows grand averaged AERPs from both study groups in response to target stimuli (digit 4) in the left ear and non-target stimuli (digits 1, 2, 3, 5, 6). The mean latency and amplitude of the N100 and P200 components did not differ significantly between the PD and control groups in response to any of these stimuli. For both study groups, the mean amplitude of the N200 component recorded in response to “target” (digit 4) stimuli was larger (greater negative voltage) than the amplitude of their N200 component in response to other (nontarget) digits. However, the N200 component elicited from control subjects in response to target digits had shorter latency (315.2 vs.343.7 msec, *p* = 0.02 for the right ear; 311.8 vs.344.6 msec, *p* = 0.009 for the left ear) and greater amplitude (*p* < 0.04 for both ears) compared to the N200 recorded from PD patients.

Because of the statistically significant difference in the proportion of males vs. females between the study groups, statistical analyses were conducted to compare AERP component latencies and amplitudes of each gender within each group. Results indicated no statistically significant differences in AERP component latencies or amplitudes recorded from male and female participants within each study group.

### 3.6. Correlations between AERP Components and Other Variables

Pearson correlation calculations were conducted to investigate and quantify the associations between AERP component latencies/amplitudes and patient characteristics, including their performance on neuropsychological and CAP assessments reported in our previous publication [12]. Results of these analyses (shown in Table 1) indicate that the latency of the N200 component was positively correlated with participant age for both the PD (*r* = 0.67, *p* = 0.00001) and control groups (*r* = 0.49, *p* < 0.003). As participant age increased, the latency of the N200 component also increased. The absolute amplitude of the N200 component was negatively correlated with participant age for both the PD and the control groups (*r* = −0.35, *p* = 0.03 in both cases). As participant age increased, the absolute amplitude of the N200 component decreased. N200 absolute amplitude was positively correlated with participants' score on the Rey Auditory Verbal Learning Test (RAVLT) [16] for both the PD (*r* = 0.39, *p* < 0.02) and control groups (*r* = 0.43, *p* < 0.01). As RAVLT score increased—indicating better cognitive functioning—the absolute amplitude of the N200 component also trended to increase. Spatial Release from Masking (SRM) scores in the 45° condition were positively correlated with N200 amplitude for both the PD group (*r* = 0.41, *p* = 0.01) and the control group (*r* = 0.46, *p* = 0.005). As SRM scores increased, N200 amplitude also tended to increase. Spatial Release from Masking (SRM) scores in the 45° condition were negatively correlated with P300 latency for both the PD group (*r* = −0.33, *p* < 0.05) and the control group (*r* = −0.35, *p* = 0.04). As SRM scores increased, P300 latency tended to decrease. Correlations between AERP components and other variables were not statistically significant.

## 4. Discussion

Behavioral assessments described in our previous publication involving these study participants did not reveal significant depression or cognitive decline for either group or significant differences in these conditions between the PD and control groups [12]. Part of the explanation for these results might be that most of the PD patients in this study were in the early or less severe stages of the disease. However, it is likely that many of these patients will experience cognitive decline as they age and their disease progresses. Unfortunately, some PD patients will also experience increases in depression for the same reasons.

### 4.1. Behavioral Assessments of Central Auditory Processing

Our previous publication involving these participants indicated that both of the study groups exhibited significant deficits in many assessments of central auditory processing (CAP), with PD patients performing worse than the control group on the 45° condition of the Spatial Release from Masking (SRM) test [12]. These results are typical for people who are >65 years of age and have significant hearing loss—characteristics which apply to many individuals in both the PD and control groups in this study. However, most of the behavioral CAP assessments used in our 2017 study did not reveal differences in performance between the study groups. This finding might be attributable to (a) insufficient sensitivity of the tests used, or (b) the fact that most of the PD patients in this study were in the early or less severe stages of the disease.

### 4.2. Audiometric Data

Audiometric results from this study are different from those published by Yýlmaz et al. [28] who reported that a group of 20 Parkinson patients had a worse hearing at 4000 and 8000 Hz compared to a group of age-matched control subjects. However, our PTA threshold results are similar to those reported by Vitale et al. [11] in a study of 106 PD patients. In that study, the PTA threshold for audiometric frequencies 0.5, 1, 2, and 4 kHz was 26 dB HL for the entire patient group, with greater degrees of hearing loss exhibited by older subgroups of participants.

### 4.3. Electrophysiological (AERP) Results—P300

Similar N100 responses to the 500 Hz nontarget tone indicate that exogenous processing of this stimulus at the level of the auditory cortex is similar for the two study groups. However, P300 mean latency in response to the 1000 Hz target tone was significantly greater for the PD group compared to the control group. Since the average hearing sensitivity of PD patients and control subjects in this study was similar for 500 and 1000 Hz tones, hearing loss was not a significant contributor to differences in P300 latency exhibited by the study groups. Neural generators of the P300 component include the frontal cortex, reticulothalamus, and the medial septal area [29, 30]—regions that play roles in attention, motivation, learning, and memory functions. Therefore, delayed P300 latency in the PD group might be an early indicator of cognitive decline or executive dysfunction in these patients [31].

In contrast to results obtained in this study, Jiang et al. [32] reported that auditory P300 amplitude was smaller in PD patients compared to healthy control subjects. However, several other AERP studies involving PD patients have shown—in agreement with our results—that the latencies of their P300 components were greater than those recorded from control subjects [32, 33]. Stanzione et al. [34] and Matsui et al. [35] reported that P300 latency was greater for older PD patients vs. younger patients with PD. Sohn et al. [36] reported that P300 latency was shorter for PD patients taking L-DOPA compared to PD patients who did not take the medication. Unfortunately, none of these studies mention (or tested?) hearing sensitivities of PD patients or control subjects. Therefore, we cannot know if the PD patients had more hearing loss than control subjects or if greater hearing loss contributed to differences in P300 components. However, Fjell and Walhovd [37] concluded that the auditory P300 remains an indicator of cognitive function even when the relationship between hearing ability and AERP generation is corrected for perceptual differences.

### 4.4. Electrophysiological (AERP) Results—Dichotic Digits

In the dichotic digits protocol, the N200 component elicited from control subjects in response to target digits had shorter latency (*p* ≤ 0.02) and greater amplitude (*p* < 0.04) compared to the N200 recorded from PD patients. This “processing negativity” in response to target stimuli reflects attentional and other executive functions employed by subjects to identify the number “4” among competing digit stimuli. Ross et al. [38] contend that both auditory and frontal cortical regions play roles in identifying target stimuli during dichotic listening tasks. Disparities in N200 components generated by PD patients (compared to age-matched control subjects) might reflect deficits in auditory processing or executive functions for the PD group*—*perhaps an early indicator of impending cognitive decline in some of these patients. In an AERP study involving Parkinson's patients, Vieregge et al. [39] reported that the processing negativity remained unchanged when patients had a 12-hour withdrawal of their usual anti-Parkinsonian drug therapy. Thus, electrophysiological results remained stable during changes in Parkinson's medication concentration within study participants.

The fact that N100 AERP components did not differ significantly between the PD and control groups in this study indicates that central auditory pathways from the cochlea to the auditory cortex functioned similarly well for both populations. However, prolonged latencies of AERP components P300 and N200 exhibited by the PD group might be early indicators of cognitive decline. For this group of PD patients, electrophysiological indicators of CAP deficits and cognitive impairment were more sensitive than behavioral CAP and neuropsychological assessments used in this study. A possible mechanism for auditory dysfunction exhibited by PD patients was suggested by Batton et al. [40]. In their study involving an animal model, the authors concluded that the subparafascicular thalamic nucleus (SPF) neuronal pathway functionally mediates dopamine release in the inferior colliculi and may be at least partially responsible for auditory processing deficits associated with PD. Based on their integrative review of published studies involving event-related potentials and cognition in Parkinson's disease, Seer et al. [31] suggested that P300 latencies and N200 amplitudes have the potential to provide biomarkers of nigrostriatal dopamine depletion associated with cognitive impairment in PD.

### 4.5. Correlations between AERP Components and Other Variables

The N200 AERP component, elicited during the dichotic digits protocol, was significantly correlated with participants' age and also with their performance on the RAVLT cognitive assessment. These correlations were statistically significant for both the PD and control groups in this study. Because the N200 component reflects higher-level processing of complex auditory stimuli, it is not surprising that its latency increased and its amplitude decreased with greater participant age. The fact that N200 absolute amplitude was positively correlated with participants' score on the RAVLT indicates that the dichotic digits protocol used in this study is a sensitive electrophysiological assessment of cognitive processing ability, perhaps more sensitive than the auditory P300 protocol, since P300 components were not significantly correlated with results of this behavioral assessment. Because the N200 was elicited by spoken digits in this study, it is possible that this component reflects some aspects of cognitive ability associated with language processing, while the P300 elicited by tonal stimuli is less likely to engage neural networks involved with language.

P300 latency and N200 amplitude were also significantly correlated with results from the SRM (45°) CAP assessment. Since the SRM 45° condition was the only behavioral CAP assessment in which the PD group exhibited significantly worse performance compared to the control group [12], it is interesting that significant correlations exist between SRM 45° scores and P300 latency/N200 amplitude. These findings support our conclusions that (a) SRM is a more sensitive CAP assessment compared to the other behavioral CAP tests used in this study; b) P300 latency and N200 amplitude are clinically useful physiological indicators that can identify cognitive and CAP processing deficits, even in early-stage PD patients. Seer et al. [31] stated that event-related potentials might be more accurate indicators of cognitive dysfunction in PD than are conventional neuropsychological tests, a conclusion which is supported by the results of this study and our previous publication involving these participants [12].

### 4.6. Limitations of This Study

Because the sample size of this study was relatively small, our conclusions regarding auditory or cognitive deficits associated with PD should be interpreted in context. An extensive battery of cognitive assessments was not included in the original study design [12]; therefore, we collected limited data on the cognitive function of participants aside from auditory processing. Also, most of the PD patients who participated in this study were in the early or less severe stages of the disease. Therefore, we do not know how more severe PD might affect auditory processing or the electrophysiological results reported here.

## 5. Conclusions

AERP assessments used in this study appear to be sensitive indicators of CAP and cognitive deficits exhibited by early-stage PD patients. While few significant differences in performance on behavioral CAP and cognitive tests were previously observed between this population of PD patients and age-matched control subjects [12], auditory N200 and P300 components recorded in the present study revealed impaired neural processing by the PD group. It is likely that many of these PD patients will exhibit additional cognitive decline and increased CAP dysfunction as they age and their disease progresses. The electrophysiological assessments described in this study could be used to evaluate and monitor the neural processing abilities of PD patients over the course of time. To our knowledge, the dichotic digits AERP protocol had not been used previously with PD patients and represents a sensitive, new physiological indicator of higher cognitive function in this population.

## Figures and Tables

**Table 1:** Pearson correlation (*r*) values for pertinent variables and assessments.

		PD group (*n* = 35)		Control group (*n* = 35)	
AERP component	Covariate	Pearson's *r*	*p*	Pearson's *r*	*p*
Latency of P300	Spatial release from masking (45°)	-0.327	<0.05	-0.348	0.04

Amplitude of N200	Participants' age	-0.354	0.03	-0.357	0.03
	Spatial release from masking (45°)	0.410	0.01	0.463	0.005
	RAVLT total score	0.392	<0.02	0.432	<0.01

Latency of N200	Participants' age	0.665	<0.00001	0.493	<0.003

## Data Availability

Study data are available on request from the authors.
